# Fibroblast Growth Factor-23—A Potential Uremic Toxin

**DOI:** 10.3390/toxins8120369

**Published:** 2016-12-08

**Authors:** Piotr Kuczera, Marcin Adamczak, Andrzej Wiecek

**Affiliations:** Department of Nephrology, Transplantation and Internal Medicine, Medical University of Silesia, Katowice 40-027, Poland; pkuczera@sum.edu.pl (P.K.); madamczak1@op.pl (M.A.)

**Keywords:** fibroblast growth factor 23, renal replacement therapy, uremia

## Abstract

Fibroblast growth factor-23 (FGF23) is a circulating member of the FGF family produced mainly by the osteocytes and osteoblasts that can act as a hormone. The main action of FGF23 is to lower phosphatemia via the reduction of urinary phosphate reabsorption and the decrease of 1,25(OH)_2_-D generation in the kidney. In the course of chronic kidney disease (CKD), plasma FGF23 concentration rises early, most probably to compensate the inability of the deteriorating kidneys to excrete an adequate amount of phosphate. However, this comes at the cost of FGF23-related target organ toxicity. Results of clinical studies suggest that elevated plasma FGF23 concentration is independently associated with the increased risk of CKD progression, occurrence of cardio-vascular complications, and mortality in different stages of CKD. FGF23 also contributes to cardiomyocyte hypertrophy, vascular calcification, and endothelial dysfunction. The impact of FGF23 on heart muscle is not dependent on Klotho, but rather on the PLCγ–calcineurin–NFAT (nuclear factor of activated T-cells) pathway. Among the factors increasing plasma FGF23 concentration, active vitamin D analogues play a significant role. Additionally, inflammation and iron deficiency can contribute to the increase of plasma FGF23. Among the factors decreasing plasma FGF23, dietary phosphate restriction, some intestinal phosphate binders, cinacalcet (and other calcimimetics), and nicotinamide can be enumerated. Anti-FGF23 antibodies have also recently been developed to inhibit the action of FGF23 in target organs. Still, the best way to normalize plasma FGF23 in maintenance hemodialysis patients is restoring kidney function by successful kidney transplantation.

## 1. Introduction

At the beginning of the 21th century it seemed that the regulation of the calcium–phosphate balance was relatively well understood. The two main “players” involved in the Ca–P homeostasis were thought to be the parathyroid hormone (PTH) and 1,25-dihydroxy-vitamin D_3_ (1,25(OH)_2_-D). Then, the discovery of fibroblast growth factor-23 (FGF23) revolutionized our understanding of Ca–P balance regulation, and changed a previous simplistic view to the complex, multi-organ feedback system that acts to maintain the physiological concentrations of calcium and phosphate. The existence and function of FGF23 was firstly hypothesized when Meyers et al. demonstrated that the “phosphate wasting factor” can be transferred from the X-linked hypophosphatemic rickets mice to normal mice in a parabiosis model [1]. Another piece of evidence emerged when a gain of function mutation for the FGF23 gene had been described in patients with autosomal hypophosphatemic rickets [2]. Further confirmation came from the studies documenting the involvement of FGF23 in the etiopathogenesis of Tumor Induced Osteomalacia (TIO) [3].

## 2. FGF23 Properties

FGF23 is an endocrine-acting 32 kDa protein mostly secreted by osteoblasts and osteocytes. It consists of 251 amino acids, of which 24 undergo limited proteolysis on secretion [3]. The *N*-terminal end has an FGF homology domain (allowing the binding to the FGF receptor), while the *C*-terminal end has a unique 72-amino acid sequence which can bind to the FGF23 coreceptor—α-Klotho. The *C*-terminal domain is also involved in the systemic action of this protein. It was shown that the lack of several amino acids in the *C*-terminal end makes this protein more soluble—mostly because of lower binding affinity to heparin [4].

It has recently been shown that the amount of FGF23 in circulation is precisely regulated by posttranslational processes. To prevent the intracellular cleavage of FGF23, it has to be *O*-glycosylated at threonine. This is clinically important, because only the intact and not-cleaved molecule exerts systemic actions; thus, the failure of this glycosylation leads to an FGF23 deficiency-like status [5]. Moreover, the aforementioned *O*-glycosylation must be counterbalanced by the phosphorylation of serine. The lack of the aforementioned process leads to the increase of circulating intact-FGF23 (iFGF23) concentration, causing a hypophosphatemic rickets-like phenotype [6]. The exact mechanisms of the regulation of posttranslational FGF23 modification are not yet fully understood; they are, however, extremely precise. The excess of *C*-terminal FGF23 (cFGF23) produced in bone in response to various (e.g., inflammatory) stimuli is counterbalanced by the augmentation of FGF23 cleavage [7]. This phenomenon leads to the stable plasma concentration of the biologically active iFGF23 [7,8]. It is however important to stress that in CKD, the cleavage process seems to be impaired, which may lead to the accumulation of cFGF23 and the increase of the cFGF23/iFGF23 ratio [8]. 

## 3. Mechanisms of Action and Toxicity

**Figure 1:**
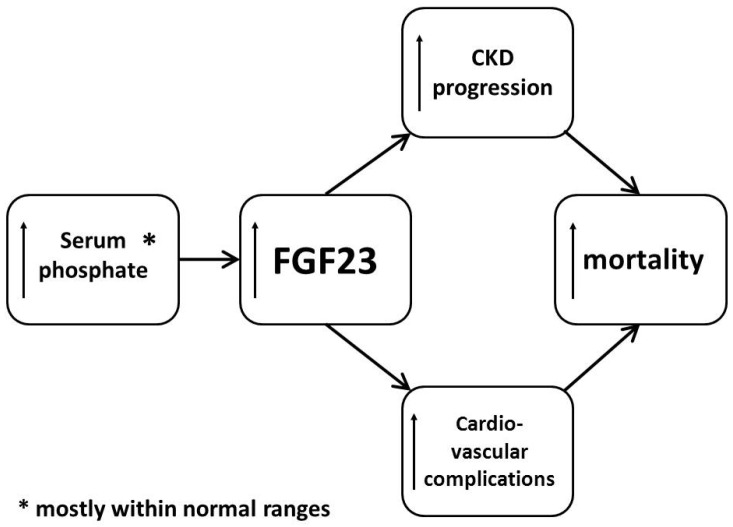
Brief summary of fibroblast growth factor-23 toxicity in chronic kidney disease. CKD—chronic kidney disease; FGF23—fibroblast growth factor-23.

Clinically, the most important site of FGF23 action (at least under physiological conditions) is the kidney. It has now been clearly documented that plasma FGF23 concentration physiologically rises in order to decrease the excess of serum phosphate [8] (Figure 1).

The FGF23-induced decrease of serum phosphate is exerted mainly through the increase of urinary phosphate excretion. This is due to the suppression of the expression of the NaPi-2a and NaPi-2c sodium-phosphate cotransporters in the apical membrane of the epithelial cells of the renal proximal tubules [9,10].

Still, the exact mechanism of the phosphaturic action of FGF23 has remained elusive for a considerable amount of time. This has arisen from the fact that in the early studies, the expression of Klotho—a mandatory FGF23 co-receptor in the kidney—has been found only in the distal renal tubules [11]. It was therefore not clear how FGF23 could act in the proximal tubules while its mandatory co-receptor was absent there. It was even hypothesized that the distal tubules may secrete some unknown paracrine-acting cytokine that would mediate the action of FGF23 in the proximal tubules in order to decrease urinary phosphate reabsorption [12]. Although this possibility has not been excluded, it has recently been observed that FGF23 can decrease the NaPi-2a expression in the renal epithelial cells in a manner resembling PTH action—through the sodium–hydrogen exchanger regulatory factor (NHERF)-1 [13]. Additionally, the apparent absence of Klotho in the proximal epithelial cells might merely be caused by the specificity of “anti-Klotho” antibodies used in the earlier studies, as Klotho expression in proximal epithelial cells has been confirmed by Andrukova et al. using different types of antibodies [13].

The second pathway in which FGF23 acts to lower serum phosphate concentration is the inhibition of 1,25(OH)_2_-D synthesis. FGF23 inhibits the synthesis of CYP27B1, which is an enzyme involved in the conversion of 25OH-D into the more active 1,25(OH)_2_-D. Moreover, FGF23 upregulates the expression of another enzyme: CYP24A1, which is essential in the catabolism of 1,25(OH)_2_-D, as well as the 25OH-D it stimulates the synthesis of 1,24,25(OH)_3_-D or 24,25(OH)_2_-D [14,15].

In addition to the phosphaturic action of FGF23, it seems that this hormone is also involved in renal calcium reabsorption through the regulation of the TRVP5 (transient receptor potential cation channel subfamily V member 5) channel in a Klotho-dependent manner [16]. This is a possible explanation of the fact that the injection of FGF23 does not lead to the development of hypocalcemia, despite the FGF23-related diminution of serum 1,25(OH)_2_-D concentration [3]. 

Another important action of FGF23 in the kidney is the conservation of sodium. This may lead to hypervolemia, and therefore, potentially to the development of hypertension [17]. This mechanism is mediated through the regulation of an abundance of NCC (sodium-chloride symporter) in the membrane of distal renal tubules. Interestingly, this effect of FGF23 was abolished by chlorothiazide administration [17].

Another major target organ for FGF23 is the parathyroid gland. FGF23 suppresses the PTH synthesis in the parathyroid gland, probably in a Klotho-dependent manner (the parathyroids are characterized by abundant expression of Klotho) [18]. However, there have been studies conducted with results suggesting otherwise. In Klotho knock-out mice, FGF23 decreased PTH production, probably by PLCγ–calcineurin–NFAT (nuclear factor of activated T-cells) pathway, and cyclosporine-A administration resulted in a lack of FGF23-dependent decrease of PTH production in these animals [19,20].

On the other hand, the excess PTH decreases the production of FGF23 in bone [21]. The mutual interactions between FGF23 and PTH can thus be seen as a typical negative feedback loop.

In CKD (chronic kidney disease) patients, plasma FGF23 concentration raises as early as in stage 2, which is much earlier than when the significant changes of phosphatemia or serum PTH concentration occur. This increase is most likely a counterbalance for the tendency to the higher (but still mostly within the physiological range) serum phosphate concentrations arising from the altered urinary phosphate excretion. Therefore, uremic toxicity of FGF23 seems to be at least partially secondary to the fledgling phosphate accumulation, and phosphate should be regarded as the “primary” uremic toxin. The decreased renal clearance of FGF23 caused by the falling GFR may also contribute to the increase of plasma FGF23 concentrations. An additional mechanism involved in the elevation of plasma FGF23 concentration in CKD is the decrease of the renal expression of Klotho concomitant with the deterioration of kidney function. This causes the renal “resistance” to circulating FGF23 [14,15,16]. An interesting recent finding is the fact that both inflammatory status and/or iron deficiency can be other potent contributors to the increased FGF23 concentration in patients with CKD. It seems that inflammation increases FGF23 release from the bone, both directly and through a mechanism related to the iron-balance with hypoxia induced factor (HIF)-1 as the main orchestrator [19].

Of note, though the plasma concentration of FGF23 is two- to five-fold above the physiological limit in the less-advanced stages of CKD, it can reach even 1000-fold above the upper limit in terminal renal failure [22,23,24,25].

As was mentioned previously, the excess of FGF23 released into the circulation allows for the maintenance of a stable serum phosphate concentration, even in an environment of declining renal function. However, this comes with a cost. Elevated plasma FGF23 concentration is independently associated with an increased risk of CKD progression, cardiovascular complications, and mortality in different stages of CKD [22,26,27,28,29,30,31]. One of the most evident studies was published in 2008. In this study, Gutierrez et al. showed the increased mortality in a large cohort (>10,000 subjects) of end-stage kidney disease (ESKD) patients beginning renal replacement treatment and followed up for one year. Elevated plasma FGF23 concentration (highest vs. lowest quartile) was associated with an almost six-fold higher risk of death [27]. In addition, in patients from the general population with coronary arteries disease, higher plasma concentrations of FGF23 have been associated with higher risk of mortality and CVD events, even after adjusting for traditional cardiovascular risk factors, serum C-reactive protein (CRP) concentrations, or renal function [32]. This was further confirmed in the LURIC (Ludwigshafen Risk and Cardiovascular Health) study, in which in patients undergoing coronary arteriography plasma FGF23 concentrations within the fourth quartile were associated with a 2.5-fold increase in all-cause and CV mortality when compared with patients with plasma FGF23 concentrations within the lowest quartile [33].

In clinical studies, high plasma FGF23 concentrations have also been associated with increased mass of left ventricle and higher prevalence of its hypertrophy, as well as the presence of vascular calcifications and dysfunction of endothelium [34,35,36,37,38,39].

The results obtained in the aforementioned studies may arise from a direct action of FGF23 on the cardiomyocytes, as this hormone has been shown to directly induce cardiomyocyte hypertrophy in both in vivo and in vitro studies [34].

Interestingly, the hypertrophic action of FGF23 on cardiomyocytes is not Klotho-dependent. It was shown by Faul et al. [34] that Klotho is virtually absent in cardiomyocytes. Nevertheless, when treated with pan-FGFR inhibitor (PD 173074), the mouse cardiomyocytes did not develop hypertrophy, regardless of the high plasma FGF23 concentration. This gives a strong assumption for the conclusion that, in fact, FGF23-induced cardiomyocyte hypertrophy is FGFR but not Klotho dependent. What is more, these authors have shown that, although FGF23 exerts its action on cardiomyocytes through FGFR, it uses a distinct specific pathway different than other FGFs. In a series of experiments, Grabner et al. recently identified FGFR4 as the target receptor for FGF23 binding in the myocardium [40]. The results of both of the aforementioned studies strongly suggest that the FGF23 signaling (after binding with FGFR4) is through the PLCγ–calcineurin–NFAT pathway rather than via the MAPK cascade (activated in a hypertrophic action of, e.g., FGF2—a classical protein stimulation of cardiac hypertrophy). This is interesting because of the possibility of attenuating the FGF23 related cardiomyocyte hypertrophy with cyclosporine-A, for example, which has already been documented by the authors in an experimental model [20]. This set of observations was then further confirmed by Touchberry et al., who showed that FGF23 significantly increases the size of adult cardiomyocyte cells [41]. Moreover, the very low expression of Klotho in the heart muscle has been confirmed in this study. It also seems likely that the final link in the pathway of FGF23’s influence on cardiomyocytes is the increase of Ca^2+^ influx to the cell, resulting in contractility changes. This action can be abolished by verapamil administration [41].

Finally, an interesting observation by Rossiant et al. must be mentioned. They have convincingly reported an involvement of FGF23 in an impairment of neutrophil recruitment and host defense in the experimental model of CKD. Briefly, as expected, CKD animals have been more prone to infection. In the experiments, the neutralization of FGF23 at different levels (e.g., with anti-FGF23 antibodies or FGF23 receptor blockade) tended to restore leukocyte recruitment into the inflammatory tissue, which was initially impaired by CKD, whereas the administration of FGF23 to control animals had the opposite effect [42].

## 4. Factors Influencing Plasma FGF23 Concentration

Results of clinical studies conducted so far have shown that increased plasma FGF23 concentration is associated with severe adverse outcomes in CKD patients. This gives a strong rationale for the attempts to lower the plasma FGF23 concentrations in patients with CKD. 

As elevated serum phosphate concentrations lead to an increase of FGF23 secretion, the most obvious approach would be to limit the intestinal phosphate delivery. The results of a small study with the application of a low-phosphate (vegetarian) diet seem to support this thesis [42]. Similar results have been obtained in patients without kidney disease [43]. The effects of a low phosphate/low protein diet on plasma concentrations of FGF23 were further confirmed by other authors [44,45,46].

Studies evaluating the effects of the administration of phosphate binders on plasma FGF23 concentrations yielded conflicting results. The use of non-calcium-based phosphate binders generally led to an approximately 30% decrease of plasma FGF23 [47,48,49]. This effect was not seen after the administration of calcium-based binders [49,50], probably because calcium is regarded as a secondary factor stimulating FGF23 synthesis [50].

Another potential method of reducing intestinal phosphate absorption is the blocking of NPT2b (sodium dependent phosphate transporter 2b). NPT2b is an intestinal phosphate transporter whose expression can be blocked by nicotinamide [51,52,53], causing the decrease of the intestinal phosphate absorption in experimental models [52] and in CKD patients [52,54]. It was shown that nicotinamide administration leads to an 11% decrease of plasma FGF23 concentration compared to placebo [55] in patients with CKD stage 3.

Vitamin D and its active metabolites are also known to modify the plasma concentrations of FGF23. As far as native vitamin D is concerned, the data presented so far is conflicting. In a study by Turner et al. [56] and Burnett-Bowie et al. [57], ergocalciferol treatment in patients with hypovitaminosis D and normal kidney function led to a considerable increase in plasma FGF23 concentration. Conversely, Uzum et al. showed that in vitamin D-deficient women with preserved renal function, cholecalciferol loading with a subsequent maintenance vitamin D_3_ dose led to a decrease in plasma FGF23 concentration (which was already low at baseline) [58]. 

The data concerning active vitamin D and its analogues is more straight-forward. It has been well documented that 1,25(OH)_2_-D increases plasma FGF23 concentrations in both rodents [59] and humans with preserved renal function [60], and the so-called active vitamin D analogues (e.g., doxecalciferol) increase the plasma concentrations of FGF23 in peritoneal dialysis patients with secondary hyperparathyroidism (sHPT) [61]. Moreover, in hemodialysis patients with sHPT, both alfacalcidol and paricalcitol have been documented to increase plasma FGF23 roughly 2–3-fold with a return to baseline values after the washout period [62]. This could give a strong rationale to not overuse active vitamin D analogues in the treatment of CKD-MBD in patients with renal failure; however, as has already been mentioned, there is some experimental data suggesting that Klotho restoration in the vasculature obtained by VDR activation with calcitriol led to the FGF23 mediated inhibition of calcification [63].

On the other hand, treatment with cinacalcet has been associated with the decrease of plasma FGF23 concentrations [64,65,66]. However, whether this is a direct influence of cinacalcet or a secondary effect of cinacalcet-related serum phosphate concentration decrease has not been univocally elucidated. Interestingly, it has already been shown that in healthy males, a single intravenous dose of a novel peptide activating the CaSR—velcaletide decreases plasma FGF23 concentration along with the serum PTH, not significantly influencing the serum phosphate concentrations [67].

Another approach is the development of anti-FGF23 antibodies. It was recently documented [68] that the administration of these antibodies in animals on a high phosphate diet somewhat reversed the CKD-related 1,25(-OH)_2_D and calcium concentration decrease, and ameliorated the severity of secondary hyperparathyroidism with the improvement of bone histomorphometry. However, this came at the cost of higher serum phosphate concentrations, with concomitant aggravated calcification of the aorta and increased incidence of death in animals treated with anti-FGF23 antibodies compared with controls. This is an interesting finding, especially in the light of results obtained by Lim et al. [63], which showed that FGF23 may in fact have some anti-calcifying properties. Nevertheless, in the first clinical studies involving patients with hypophosphatemic rickets/osteomalacia, the use of anti-FGF23 antibodies gave promising results [68,69,70].

As was mentioned previously, there are lines of evidence emerging that suggest a role of inflammation and/or iron deficiency in the increased production of FGF23 in bone. Nevertheless, the exact underlying mechanisms remain somewhat elusive. It seems that a main player is the hypoxia-inducible factor-1a (HIF-1a), which is involved in the regulation of FGF23 cleavage processes. Besides HIF-1a, the direct inflammatory signals (e.g., interleukin-1) also stimulate the FGF23 production [19,71]. Interestingly, it seems that the acute inflammation and functional iron deficiency lead only to an increase of cFGF23, while the plasma concentration of iFGF23 remains stable. This is probably due to the augmentation of FGF23 cleavage, and results in normal concentrations of the biologically active form—iFGF23 [7,71,72]. However, this is only true in subjects with preserved renal function. In animals with CKD, IL-1β injection resulted in an increase of both cFGF23 and iFGF23 concentrations [72]. In addition, chronic inflammation can lead to elevated plasma concentrations of iFGF23, which is probably caused by the alteration of FGF23 production and cleavage balance [72].

However tempting the theory is, no studies have been conducted so far implying that the limitation of inflammation or the correction of iron deficiency could decrease the elevated plasma FGF23 concentrations in CKD. Conversely, the injection of iron polymaltose caused an increase in plasma FGF23 concentration, concomitantly with the decrease of serum phosphate concentration in patients with iron deficiency and preserved kidney function [73].

Finally, it should be noted that successful kidney transplantation usually tends to lower the markedly increased FGF23 concentrations so often seen in advanced stages of CKD [74,75,76,77]. The results of some clinical studies suggest that despite the rapid decline in plasma FGF23 concentration after kidney transplantation, it is still slightly but significantly higher than in the general population [77]. This could be attributed to the common use of phosphate supplementation or active vitamin D treatment of the post-transplant hypophosphatemia [74,78], but may also be related to the observations that calcineurin inhibitors and sirolimus in fact stimulate FGF23 production [75,76]. On the other hand, no differences in the plasma concentration of FGF23 between the post-transplant and CKD population with equal eGFR have been found [74].

In conclusion: Plasma FGF23 concentration rises early in the course of chronic kidney disease, most probably to compensate the inability of the deteriorating kidneys to excrete an adequate amount of phosphate. In addition, the results of clinical studies suggest that elevated plasma FGF23 concentration is independently associated with an increased risk of CKD progression, occurrence of cardio-vascular complications, and mortality in different stages of CKD. These facts give a strong rationale for regarding FGF23 as a uremic toxin.
